# Supplementary material for: Meta-Analysis and Gene Set Enrichment Relative to ER Status Reveal Elevated Activity of MYC and E2F in the “Basal” Breast Cancer Subgroup
Source: PLoS One. 2009 Mar 9;4(3):e4710. doi: 10.1371/journal.pone.0004710 (PMC2650420; doi:10.1371/journal.pone.0004710)
Supplement: Dataset S1 — GSEA results from first screen. The file contains a zipped archive of the HTML results of the second GSEA screen. Reports for enriched or depleted datasets may be accessed from the “index.html” link within the my_analysis.GseaPreranked.1219975950625.rpt.zip ZIP archive. Enrichment plots are available for each of the 50 most enriched and depleted gene sets. (13.32 MB ZIP) [file pone.0004710.s001.zip › my_analysis.GseaPreranked.1219975950625.rpt.zip!//CHR5Q31.html]

Details for gene set CHR5Q31[GSEA]

|  || Dataset | zSco.ERneg.to.ERpos\_collapsed |
| Phenotype | NoPhenotypeAvailable |
| Upregulated in class | na\_neg |
| GeneSet | CHR5Q31 |
| Enrichment Score (ES) | -0.42270863 |
| Normalized Enrichment Score (NES) | -2.0023844 |
| Nominal p-value | 0.0 |
| FDR q-value | 0.00906967 |
| FWER p-Value | 0.189 |
Table: GSEA Results Summary

  

Fig 1: Enrichment plot: CHR5Q31      
 Profile of the Running ES Score & Positions of GeneSet Members on the Rank Ordered List

  

| PROBE | GENE SYMBOL | GENE\_TITLE | RANK IN GENE LIST | RANK METRIC SCORE | RUNNING ES | CORE ENRICHMENT || 1 | KIF20A |  |  | 318 | 4.874 | -0.0037 | No |
| 2 | TCOF1 |  |  | 631 | 4.075 | -0.0103 | No |
| 3 | ACSL6 |  |  | 852 | 3.705 | -0.0115 | No |
| 4 | PDE6A |  |  | 1198 | 3.236 | -0.0241 | No |
| 5 | TGFBI |  |  | 1668 | 2.786 | -0.0480 | No |
| 6 | CDC25C |  |  | 1794 | 2.700 | -0.0462 | No |
| 7 | NPY6R |  |  | 1836 | 2.664 | -0.0381 | No |
| 8 | VDAC1 |  |  | 2049 | 2.484 | -0.0438 | No |
| 9 | TCF7 |  |  | 2067 | 2.473 | -0.0347 | No |
| 10 | PDLIM4 |  |  | 2068 | 2.472 | -0.0244 | No |
| 11 | PCDHB1 |  |  | 2117 | 2.444 | -0.0178 | No |
| 12 | ARHGAP26 |  |  | 2178 | 2.399 | -0.0123 | No |
| 13 | PCDHGC3 |  |  | 2277 | 2.334 | -0.0100 | No |
| 14 | DIAPH1 |  |  | 2307 | 2.316 | -0.0025 | No |
| 15 | HTR4 |  |  | 2655 | 2.115 | -0.0199 | No |
| 16 | CENTD3 |  |  | 2845 | 2.016 | -0.0258 | No |
| 17 | PCDHA10 |  |  | 3025 | 1.921 | -0.0314 | No |
| 18 | MYOT |  |  | 3112 | 1.882 | -0.0300 | No |
| 19 | CDX1 |  |  | 3259 | 1.814 | -0.0335 | No |
| 20 | PCDHGA3 |  |  | 3334 | 1.779 | -0.0316 | No |
| 21 | GLRA1 |  |  | 3348 | 1.773 | -0.0252 | No |
| 22 | PCDHGA10 |  |  | 3375 | 1.760 | -0.0198 | No |
| 23 | SLC26A2 |  |  | 3673 | 1.630 | -0.0355 | No |
| 24 | PCDHB17 |  |  | 3914 | 1.547 | -0.0472 | No |
| 25 | FAM53C |  |  | 3920 | 1.545 | -0.0411 | No |
| 26 | PCDHGB5 |  |  | 4032 | 1.500 | -0.0433 | No |
| 27 | PCDHGA9 |  |  | 4545 | 1.305 | -0.0766 | No |
| 28 | SMAD5 |  |  | 4732 | 1.242 | -0.0855 | No |
| 29 | SPINK5 |  |  | 4859 | 1.203 | -0.0900 | No |
| 30 | PCDHGA1 |  |  | 4880 | 1.195 | -0.0866 | No |
| 31 | REEP2 |  |  | 4911 | 1.186 | -0.0839 | No |
| 32 | LRRTM2 |  |  | 5098 | 1.116 | -0.0933 | No |
| 33 | DAMS |  |  | 5245 | 1.065 | -0.0999 | No |
| 34 | PCDHA9 |  |  | 5383 | 1.019 | -0.1060 | No |
| 35 | PPP2R2B |  |  | 5785 | 0.882 | -0.1327 | No |
| 36 | ITK |  |  | 5843 | 0.863 | -0.1334 | No |
| 37 | BRD8 |  |  | 6010 | 0.808 | -0.1426 | No |
| 38 | DND1 |  |  | 6090 | 0.785 | -0.1454 | No |
| 39 | PURA |  |  | 6111 | 0.780 | -0.1436 | No |
| 40 | IL9 |  |  | 6114 | 0.780 | -0.1405 | No |
| 41 | PCDHA2 |  |  | 6153 | 0.764 | -0.1402 | No |
| 42 | RPS14 |  |  | 6165 | 0.761 | -0.1378 | No |
| 43 | PCDHGA11 |  |  | 6507 | 0.647 | -0.1610 | No |
| 44 | SLC6A7 |  |  | 6620 | 0.615 | -0.1669 | No |
| 45 | TRPC7 |  |  | 6624 | 0.614 | -0.1645 | No |
| 46 | PCDHB11 |  |  | 6698 | 0.588 | -0.1676 | No |
| 47 | LECT2 |  |  | 6747 | 0.574 | -0.1688 | No |
| 48 | IL12B |  |  | 6837 | 0.540 | -0.1733 | No |
| 49 | ALDH7A1 |  |  | 6884 | 0.524 | -0.1746 | No |
| 50 | CD74 |  |  | 7061 | 0.463 | -0.1860 | No |
| 51 | IL13 |  |  | 7152 | 0.435 | -0.1910 | No |
| 52 | TCERG1 |  |  | 7338 | 0.369 | -0.2035 | No |
| 53 | GFRA3 |  |  | 7384 | 0.350 | -0.2055 | No |
| 54 | ETF1 |  |  | 7421 | 0.338 | -0.2068 | No |
| 55 | PCDHB6 |  |  | 7553 | 0.288 | -0.2155 | No |
| 56 | AFF4 |  |  | 7673 | 0.239 | -0.2235 | No |
| 57 | WDR55 |  |  | 7881 | 0.153 | -0.2386 | No |
| 58 | NDUFA2 |  |  | 8200 | 0.035 | -0.2625 | No |
| 59 | PCDHGB6 |  |  | 8227 | 0.024 | -0.2644 | No |
| 60 | GM2A |  |  | 8232 | 0.022 | -0.2646 | No |
| 61 | POU4F3 |  |  | 8264 | 0.011 | -0.2669 | No |
| 62 | SEC24A |  |  | 8292 | 0.003 | -0.2689 | No |
| 63 | CTNNA1 |  |  | 8309 | -0.001 | -0.2701 | No |
| 64 | ANKHD1 |  |  | 8331 | -0.007 | -0.2717 | No |
| 65 | FSTL4 |  |  | 8348 | -0.015 | -0.2728 | No |
| 66 | PCDHB3 |  |  | 8421 | -0.045 | -0.2781 | No |
| 67 | HINT1 |  |  | 8664 | -0.157 | -0.2958 | No |
| 68 | UBE2D2 |  |  | 8776 | -0.199 | -0.3034 | No |
| 69 | PCDHB8 |  |  | 8781 | -0.200 | -0.3029 | No |
| 70 | HSPA4 |  |  | 8813 | -0.214 | -0.3043 | No |
| 71 | CDC42SE2 |  |  | 8826 | -0.222 | -0.3043 | No |
| 72 | PCDHA5 |  |  | 8836 | -0.225 | -0.3040 | No |
| 73 | FGF1 |  |  | 8966 | -0.297 | -0.3126 | No |
| 74 | SPRY4 |  |  | 9093 | -0.359 | -0.3206 | No |
| 75 | PCDHB12 |  |  | 9447 | -0.538 | -0.3451 | No |
| 76 | PCDHB13 |  |  | 9461 | -0.544 | -0.3438 | No |
| 77 | PCDHA3 |  |  | 9705 | -0.672 | -0.3594 | No |
| 78 | PITX1 |  |  | 9806 | -0.728 | -0.3640 | No |
| 79 | HNRPA0 |  |  | 9885 | -0.775 | -0.3666 | No |
| 80 | TMCO6 |  |  | 10096 | -0.906 | -0.3788 | No |
| 81 | PCDH12 |  |  | 10196 | -0.956 | -0.3823 | No |
| 82 | CNOT8 |  |  | 10199 | -0.957 | -0.3784 | No |
| 83 | PCDHA6 |  |  | 10208 | -0.962 | -0.3750 | No |
| 84 | P4HA2 |  |  | 10386 | -1.085 | -0.3839 | No |
| 85 | NR3C1 |  |  | 10670 | -1.297 | -0.3999 | No |
| 86 | HDAC3 |  |  | 10972 | -1.548 | -0.4162 | Yes |
| 87 | PKD2L2 |  |  | 10978 | -1.549 | -0.4101 | Yes |
| 88 | MATR3 |  |  | 11061 | -1.627 | -0.4095 | Yes |
| 89 | PFDN1 |  |  | 11107 | -1.669 | -0.4060 | Yes |
| 90 | ADRB2 |  |  | 11132 | -1.689 | -0.4007 | Yes |
| 91 | PHF15 |  |  | 11215 | -1.755 | -0.3996 | Yes |
| 92 | C5ORF4 |  |  | 11268 | -1.803 | -0.3960 | Yes |
| 93 | YIPF5 |  |  | 11377 | -1.909 | -0.3962 | Yes |
| 94 | SPARC |  |  | 11451 | -1.990 | -0.3934 | Yes |
| 95 | SLC22A4 |  |  | 11600 | -2.124 | -0.3957 | Yes |
| 96 | JMJD1B |  |  | 11707 | -2.230 | -0.3944 | Yes |
| 97 | HSPA9B |  |  | 11777 | -2.299 | -0.3900 | Yes |
| 98 | CDKL3 |  |  | 11923 | -2.466 | -0.3907 | Yes |
| 99 | CDC23 |  |  | 11927 | -2.473 | -0.3806 | Yes |
| 100 | HARS |  |  | 11935 | -2.480 | -0.3707 | Yes |
| 101 | KIAA0141 |  |  | 12187 | -2.824 | -0.3779 | Yes |
| 102 | SIL1 |  |  | 12323 | -2.992 | -0.3757 | Yes |
| 103 | SRA1 |  |  | 12393 | -3.086 | -0.3680 | Yes |
| 104 | KLHL3 |  |  | 12401 | -3.104 | -0.3555 | Yes |
| 105 | DDX46 |  |  | 12416 | -3.125 | -0.3435 | Yes |
| 106 | H2AFY |  |  | 12443 | -3.171 | -0.3322 | Yes |
| 107 | SAR1B |  |  | 12598 | -3.430 | -0.3295 | Yes |
| 108 | NDFIP1 |  |  | 12621 | -3.472 | -0.3167 | Yes |
| 109 | APBB3 |  |  | 12654 | -3.529 | -0.3043 | Yes |
| 110 | TAF7 |  |  | 12660 | -3.542 | -0.2899 | Yes |
| 111 | SPOCK1 |  |  | 12719 | -3.653 | -0.2790 | Yes |
| 112 | C5ORF5 |  |  | 12738 | -3.711 | -0.2648 | Yes |
| 113 | C5ORF14 |  |  | 12815 | -3.921 | -0.2542 | Yes |
| 114 | SEPP1 |  |  | 12828 | -3.949 | -0.2386 | Yes |
| 115 | SEPT8 |  |  | 12833 | -3.965 | -0.2223 | Yes |
| 116 | PDGFRB |  |  | 12886 | -4.104 | -0.2091 | Yes |
| 117 | IK |  |  | 12908 | -4.168 | -0.1932 | Yes |
| 118 | CXCL14 |  |  | 12915 | -4.187 | -0.1761 | Yes |
| 119 | C5ORF15 |  |  | 12937 | -4.282 | -0.1598 | Yes |
| 120 | UQCRQ |  |  | 12939 | -4.284 | -0.1420 | Yes |
| 121 | SKP1A |  |  | 13021 | -4.528 | -0.1292 | Yes |
| 122 | ZCCHC10 |  |  | 13029 | -4.548 | -0.1107 | Yes |
| 123 | RAPGEF6 |  |  | 13045 | -4.625 | -0.0924 | Yes |
| 124 | KIF3A |  |  | 13102 | -4.823 | -0.0765 | Yes |
| 125 | DCTN4 |  |  | 13113 | -4.875 | -0.0569 | Yes |
| 126 | HARSL |  |  | 13120 | -4.912 | -0.0368 | Yes |
| 127 | C5ORF3 |  |  | 13187 | -5.310 | -0.0195 | Yes |
| 128 | SLC22A5 |  |  | 13310 | -7.063 | 0.0008 | Yes |
Table: GSEA details [plain text format]

  

Fig 2: CHR5Q31: Random ES distribution      
 Gene set null distribution of ES for **CHR5Q31**
